# Localized Surface Plasmon Resonance Dependence on Misaligned Truncated Ag Nanoprism Dimer

**DOI:** 10.1186/s11671-017-2062-4

**Published:** 2017-06-30

**Authors:** Hanning Yang, Edgar Oduor Owiti, Xiangqian Jiang, Siren Li, Peng Liu, Xiudong Sun

**Affiliations:** 1 Institute of Modern Optics, Department of Physics, Harbin Institute of Technology, Harbin, 150001 China; 2 Key Laboratory of Micro-Nano Optoelectronic Information System of Ministry of Industry and Information Technology, Harbin, 150001 China; 3 Key Laboratory of Micro-Optics and Photonic Technology of Heilongjiang Province, Harbin Institute of Technology, Harbin, 150001 China; 4 Collaborative Innovation Center of Extreme Optics, Shanxi University, Taiyuan, 030006 Shanxi People’s Republic of China

**Keywords:** LSPR, Misalignment, Truncation, Ag Nanoprism dimer

## Abstract

Misaligned edge-to-edge dimers are the common products during the preparation of Ag nanoprism dimers using self-assembly method. However, in the self-assembly method, Ag nanoprisms are easily truncated because they are easy to oxidize in an acidic environment. In this work, modeling a truncated Ag nanoprism on a misaligned edge-to-edge dimer provides a better understanding of the effects of the truncation and misalignment on localized surface plasmon resonance (LSPR) of the dimer. The resonant wavelength and intensity of the dimer are flexibly modulated by changing the misalignment length of the dimer. As the misalignment length increases, a stronger peak at the shorter wavelength and a weaker one at the longer wavelength are observed. The resonant wavelengths and intensities of the two peaks are also flexibly tuned by adjusting the truncated length of the Ag nanoprism in the dimer. The results are numerically demonstrated based on the finite element method (FEM) and show promising potential for nanoswitch, multi-channel tunable biosensor and other nanodevice applications.

## Background

Silver and gold nanoparticles have attracted extensive attentions due to their unique optical properties which originate from their localized surface plasmon resonance (LSPR) effects. The conduction electrons in these nanoparticles collectively oscillate with the incident light causing an enhanced electric field that is localized around the surface of the nanoparticle [1, 2]. The LSPR effect of nanoparticles can be modulated by changing the nanoparticle’s size, shape, material, and the surrounding environment [3–5]. Among the numerous nanoparticles, nanoprisms (NPs) have attracted the most attention due to their anisotropic geometrical properties and tunable LSPR properties [6–8]. Because of the anisotropic geometrical properties, oscillating charges on the surface tend to accumulate around the NP’s tips making the localized electric field around NP more enhanced than in isotropic nanoparticles [9]. The LSPR properties of NP are usually divided into in-plane dipolar resonance, in-plane quadrupolar resonance, and out-of-plane dipolar resonance. The in-plane dipolar resonance shows a redshift effect with increasing the edge length of NP [10, 11].

As two nanoparticles are brought close to each other, the enhanced electric field concentrates in the gap of the dimer. This phenomenon is known as hot spot effect which originates from the LSPR coupling effect between the nanoparticles. The dimers with remarkable hot spot effects can be easily prepared by e-beam lithography [12], nanosphere lithography [13], and self-assembly method [14, 15]. For the self-assembly method, metal nanoparticles with specific geometries are linked through the molecules with particular groups. Therefore, the gap in the dimer is as narrow as the molecular chain length. This further reduces the gap between two nanoparticles and causes a more remarkable hot spot effect [16–18].

The hot spot effect of the NP dimer as a typical dimer has been extensively studied, but most studies about the NP dimers concentrate on the tip-to-tip geometry [19–23]. However, during preparation process of the tip-to-tip dimer through the self-assembly method, there is a wide existence of NP dimers with edge-to-edge geometries [15]. The self-assembly method often causes a randomly misaligned effect for the NP dimers. The randomly misaligned edge-to-edge Au NP dimers with the broken symmetry can induce a novel optical property [24]. This result indicates that the double resonances can be switched by modulating the misalignment length of the dimer. The resonant positions can be tuned by changing the gap length and the thickness of Au NP. Due to the sensitivity of optical properties on the structural parameters, this finding paves a promising way for developing nanoswitches, nanomotors, nanorulers, and dual-channel biosensors. However, because of the inertia of the gold atom, the structural parameters of Au NP dimers are usually fixed once they are prepared.

Because Ag is easily oxidized, the tips of the Ag NP can be easily etched in the process of its preparation. This results to Ag NP transforming into an Ag truncated nanoprism (TNP). The change of the structural symmetry originating from the truncation effect induces some novel optical properties [8, 25]. The truncation effect can be introduced in the misaligned edge-to-edge Ag NP dimer to obtain a tunable device with novel optical properties. Here, effects of the truncation and misalignment on the LSPR edge-to-edge Ag TNP dimers are studied using FEM.

## Methods

### Structural Model of Misaligned Truncated Ag Nanoprism Dimer

**Fig. 1:**
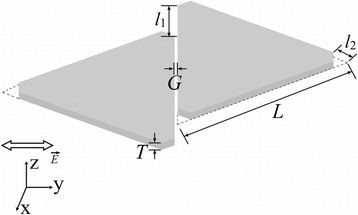
Schematic diagram of the Ag TNP dimer showing a specific misalignment length *l*_1_

The structural model used in the calculations is shown in Fig. 1. Ag NPs used in the self-assembly method are usually prepared by the seed-induced growth method: its edge length is flexibly modulated by controlling the reaction conditions and keeping the thickness constant [26]. In this paper, the thickness of Ag NP is kept constant at *T* = 8 nm. The Ag NP edge-to-edge dimers are assumed to be prepared by the self-assembly method; therefore, the gap length in this dimer (*G*) has a specific molecular length *G* = 2 nm.

The misaligned edge-to-edge dimer consists of two identical Ag TNPs with the following dimensions, edge length (*L*), misalignment length (*l*_1_), and truncated length (*l*_2_). The tips of Ag NP are cut off along a straight line with the initial edge length *L* = 130 nm and truncated length *l*_2_ = 10 nm. To study the effect of the misalignment, a misalignment ratio *R* = *l*_1_/*L* of Ag TNP dimer is varied from 0 to 1.5. As *R* approached 0 or 1, the Ag TNP dimers with the increased truncated length were modeled to study the influence of the truncation effect. Ag TNP transforms to a hexagonal nanoplate (HNP), as the edge length of Ag HNP (*L*_*1*_) is equal to the truncated length *l*_2_ = *L*/3. To further investigate the effect of the truncation and misalignment, Ag HNP dimers with the misalignment ratio *R*_*1*_ = *l*_1_/*L*_*1*_, ranging from 0 to 3, were simulated.

### Finite Element Method for Misaligned Truncated Ag Nanoprism Dimer

FEM method using COMSOL Multiphysics is used to investigate the effect of misalignment length on the LSPR edge-to-edge Ag TNP dimer. The relative permittivity for Ag was obtained from the Drude model, *ε*(*ω*) = *ε*_∞_ − *ω*_*p*_^2^/[*ω*(*ω* + *iγ*)], where *ε*_∞_ = 3.7 is the infinite frequency, *ω*_*p*_ = 1.38 × 10^16^ is the bulk plasma frequency, and *γ* = 3.72 × 10^13^ is the oscillation damping of electrons [27].

The Ag TNP dimer was modeled on the *x-y* plane at *z* = 0 with air (*n* = 1) surrounding it. Using air around the dimer was done to simplify the computation process. The incident light, polarized along the *y*-axis, was directed normally along *z*-axis on the Ag TNP surface. Tuning of the wavelength was done between *λ* = 600 nm to *λ* = 1100 nm with a step of 4 nm.

## Results and Discussion

**Fig. 2:**
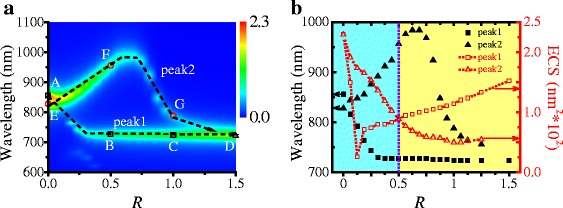
ECS spectra of Ag TNP dimers. **a** ECS intensity distribution map as functions of *R* and wavelength. **b** ECS resonant wavelength (the *dark scatter line*) and intensity (the *red short dotted line*) spectra versus *R*. The *square* and *triangle lines* show peak 1 and peak 2, respectively

The extinction cross-section (ECS) intensity distribution map of the Ag TNP dimer showing variation between the wavelength and *R* was firstly calculated to investigate effect of the misalignment length on the LSPR. Figure 2 shows the ECS intensity distribution color map of the Ag TNP dimer. As shown in Fig. 2, the Ag TNP dimer with *R* = 0 displays two peaks with equal intensities. When *R* is equal to 1, the shorter wavelength peak, labeled as peak 1, is strong and shows a blueshift effect. For the longer wavelength, labeled as peak 2, the intensity decreases and shows a blueshift effect. As *R* is increased from 0 to 1, the resonant wavelength of peak 1 initially decreases and then slightly stabilizes. Meanwhile, its intensity gradually increases. It is worth noting that peak 1 decreases abruptly when *R* changes from 0 to 1/8. This is because the disappearance of the higher-order LSPR mode. The resonant wavelength of peak 2 first increases and then decreases, but its intensity decreases throughout.

Peak 1 in Fig. 2a strengthens further as *R* increases from 1 to 1.5 and its resonant wavelength is kept unchanged. Moreover, peak 2 continuously decreases until it disappears while its resonant wavelength decreases throughout. As *R* reaches 1.5, the double peaks degenerate into a single peak with a resonant wavelength equal to that of the Ag TNP monomer. When *R* is large than 1, the LSPR coupling interaction between Ag TNPs is extremely weak. In Fig. 2b, peak 2 plays the dominant role in ECS spectrum when *R* < 0.5 (the blue region) whereas peak 1 dominates when *R* > 0.5 (the yellow region). Therefore, the two peaks can be switched on or off by modulating the misalignment length of the dimer.

**Fig. 3:**
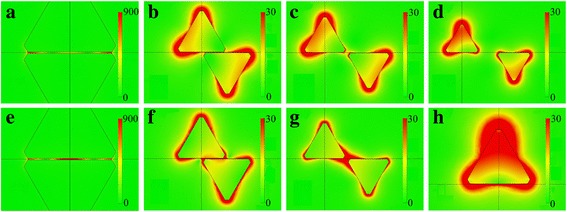
Calculated electric field distributions of Ag TNP dimers and monomer. Images are taken as the cross-section of the Ag TNP dimer at *z* = 0 on the *x-y* plane. Panels **a**–**g** correspond to the points A–G marked in Fig. 2a. Peak 1: **a** *R* = 0; **b** *R* = 0.5, B; **c** *R* = 1; **d** *R* = 1.5. Peak 2: **e** *R* =0, **f** *R* = 0.5, **g** *R* = 1. **h** Monomer

The electric field distributions of Ag TNP dimers for different values of *R* are shown in Fig. 3. The geometrical configuration of the Ag TNP dimer changes as *R* varies from 0 to 1.5. When *R* is equal to 0, the Ag TNP dimer possesses the edge-to-edge geometry. As *R* reaches to 1, the configuration changes into the tip-to-tip geometry, and as *R* increases further, the configuration turns an abnormal tip-to-tip geometry. In Fig. 3a, the enhanced electric fields are localized at the center and two ends of the gap, so for *R* = 0, peak 1 is a higher-order LSPR mode. When *R* is larger than 0, the higher-order LSPR mode disappears and the structural symmetry of the dimer is broken. Then peak 1 can be attributed to the antisymmetric mode from the LSPR coupling interaction between Ag truncated nanoprisms in the dimer shown in Fig. 3b–d. The electric fields mainly distribute around the lateral tips away from the gap in the Ag TNP dimer. When *R* is large enough, the electric field distributions of Ag TNPs in the dimer are similar to that of the Ag TNP monomer as shown in Fig. 3h. Figure 3e–g show that peak 2 can be attributed to the symmetric mode from the LSPR coupling interaction between Ag truncated nanoprisms in the dimer. The enhanced electric fields are mainly localized in the gaps. The results show that the two peaks can be turned on or off by modulating the LSPR coupling interaction between Ag TNPs in the dimer. When *R* is equal to 0, Ag TNP dimers for the two peaks possess huge enhanced electric fields (above 900 V/m) in the gap. The enhancement effect of the surrounding electric field of the Ag TNP dimers weakens as *R* increases. This means that the misalignment effect will weaken the hot spot effect of Ag TNP dimer, if *R* increases and the enhanced electric field distribution will shift from the gap to the lateral tips of Ag TNP in the dimer.

**Fig. 4:**
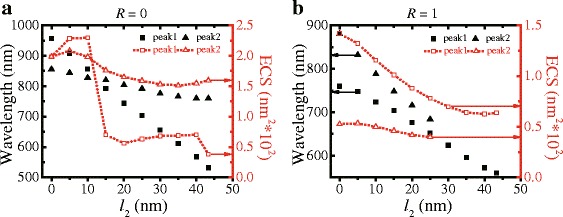
ECS spectra of Ag TNP dimers with *l*_2_ changing from 0 to 43.3 nm. **a** *R* = 0; **b** *R* = 1. The *dark scatter* and *red short dot lines* show the resonant wavelength and intensity spectra, respectively. The *square* and *triangle lines* show peak 1 and peak 2, respectively

To investigate the effect of the truncation on the switching, the truncated length-dependent ECS resonant wavelength and intensity spectra of the Ag TNP dimers are simulated and shown in Fig. 4 (for *R* = 0, 1). When the truncated length *l*_2_ is 43.3 nm and *L* = 130 nm, Ag TNP turns into Ag HNP. As shown in Fig. 4a, peak 1 abruptly decreases from the initial strong state and then stabilizes in a weak state as *l*_2_ is increased when *R* = 0. This is because the higher-order LSPR mode corresponding to peak 1 at the same *R* value no longer exists in the gap as the gap length decreases (i.e., as *l*_2_ increases). The origin of peak 1 changes from the higher-order LSPR mode to the antisymmetric mode of the LSPR coupling interaction as the truncated length increases. When *R* equals to 0 or 1 as shown in Fig. 4, the resonant wavelengths of the two peaks decrease throughout. As shown in Fig. 4b, peak 2 gradually fades as *l*_2_ increases to a specific value, the double peaks degenerate to a single peak. Similarly, this is due to the symmetric mode from the LSPR coupling interaction weakening as the interval between the two Ag TNPs increases (i.e., as *l*_2_ increases). This means that the switching effect of the Ag TNP dimer can be flexibly modulated by changing the truncated length originating from the easy-to-oxidize features of Ag.

**Fig. 5:**
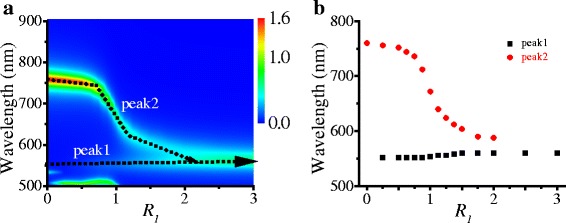
ECS spectra of the Ag HNP dimer. **a** ECS intensity distribution map versus *R*_1_ and wavelength. **b** Resonant wavelength spectra of the two peaks

When the truncated length increases to a critical value, Ag TNP in the dimer transforms into Ag HNP. The edge length of Ag HNP *L*_*1*_ is 43.3 nm. Figure 5 shows the ECS intensity distribution map of Ag HNP dimers versus *R*_1_ and wavelength. Here, the wavelength of the incident light ranges from 500 to 900 nm. For the Ag HNP edge-to-edge dimer, a change of the misalignment length (*l*_1_) also causes the variations of two peaks in its ECS spectrum. With increasing the misalignment ratio *R*_*1*_ = *l*_1_/*L*_*1*_, the switching effect of the Ag HNP edge-to-edge dimer is more remarkable than the Ag TNP dimer. Peak 2 originating from the symmetric mode of the LSPR coupling interaction quickly fades in the ECS spectrum with the two Ag HNPs moving away from each other (*R*_*1*_ > 1) as shown in Fig. 5. Peak 1 originates from the antisymmetric mode from the LSPR coupling interaction between Ag HNPs and gradually occupies the dominant role. The intensity ratio of peak 2 to peak 1 for the Ag HNP dimer is obviously larger than that of the Ag TNP dimer. Compared with the ECS spectrum of the Ag TNP dimer, the Ag HNP dimer possesses a single peak configuration which also can be switched by changing the misalignment length. This is further evidence that Ag NP edge-to-edge dimers with different truncated length possess a misalignment-related switching effect.

## Conclusions

In summary, the LSPR effects of Ag TNP misaligned edge-to-edge dimers have been studied using FEM. The ECS spectrum of Ag TNP dimer possesses two peaks, which can be switched on or off by modulating the misalignment length. When *R* is less than 0.5, the longer wavelength peak plays the prominent role in the ECS spectrum. As *R* grows larger than 0.5, the shorter wavelength peak occupies the dominant role. Due to the truncation effect, the resonant wavelengths of the two peaks can be flexibly modulated by changing the truncated length. When the truncated length increases to a specific value, Ag TNP transforms into Ag HNP. The double peaks degenerate to a single peak, and the peaks of the Ag HNP dimer also can be switched by changing the misalignment length. The calculated results indicate that Ag TNP misaligned edge-to-edge dimers pave the way for a promising surface-enhanced Raman spectrum, nanoswitch, multi-channel tunable biosensor, other nanodevices, etc.

## Abbreviations

ECS: Extinction cross-section
FEM: Finite element method
*G*: Gap length
HNP: Hexagonal nanoplate
*L*: Edge length
*l*_1_: Misalignment length
*l*_2_: Truncated length
LSPR: Localized surface plasmon resonance
NP: Nanoprism
*R*: Misalignment ratio
*T*: Thickness
TNP: Truncated nanoprism
